# Cellular and Molecular Events that Occur in the Oocyte during Prolonged Ovarian Storage in Sheep

**DOI:** 10.3390/ani10122414

**Published:** 2020-12-17

**Authors:** Alicia Martín-Maestro, Irene Sánchez-Ajofrín, Carolina Maside, Patricia Peris-Frau, Daniela-Alejandra Medina-Chávez, Beatriz Cardoso, José Carlos Navarro, María Rocío Fernández-Santos, José Julián Garde, Ana Josefa Soler

**Affiliations:** SaBio IREC (CSIC-UCLM-JCCM), ETSIAM, Campus Universitario, s/n, 02071 Albacete, Spain; alicia.martinmaestro@uclm.es (A.M.-M.); carolina.maside@uclm.es (C.M.); patricia.peris@uclm.es (P.P.-F.); daniela.medina@uclm.es (D.-A.M.-C.); beacardoso_14@hotmail.com (B.C.); jnavarropedrosa@gmail.com (J.C.N.); mrocio.fernandez@uclm.es (M.R.F.-S.); julian.garde@uclm.es (J.J.G.)

**Keywords:** sheep, ovary storage, transport, oocyte, in vitro embryo production

## Abstract

**Simple Summary:**

Establishing efficient in vitro embryo production (IVP) protocols in sheep usually requires prolonged transportation of post-mortem ovaries since adult animals are often slaughtered in abattoirs far from laboratories. In this study, different analyses were carried out to investigate important cellular and molecular aspects of hypoxic injury on excised ovaries over time in order to understand the factors jeopardizing the development of competent oocytes during prolonged transport times. We observed that, when ovaries were stored for more than 7 h, the quality and developmental potential of oocytes and cumulus cells were greatly reduced. Moreover, the use of medium TCM199 over saline solution also had deleterious effects. Beyond transport time, strategies aimed at reducing these damages may improve oocyte quality and developmental competence.

**Abstract:**

For the past two decades, there has been a growing interest in the application of in vitro embryo production (IVP) in small ruminants such as sheep. To improve efficiency, a large number abattoir-derived ovaries must be used, and long distances from the laboratory are usually inevitable when adult animals are used. In that scenario, prolonged sheep ovary transportation may negatively affect oocyte developmental competence. Here, we evaluated the effect of ovary storage time (3, 5, 7, 9, 11 and 13 h) and the medium in which they were transported (TCM199 and saline solution) on oocyte quality. Thus, live/dead status, early apoptosis, DNA fragmentation, reduced glutathione (GSH) and reactive oxygen species (ROS) content, caspase-3 activity, mitochondrial membrane potential and distribution, and relative abundance of mRNA transcript levels were assessed in oocytes. After in vitro maturation (IVM), cumulus cell viability and quality, meiotic and fertilization competence, embryo rates and blastocyst quality were also evaluated. The results revealed that, after 7 h of storage, oocyte quality and developmental potential were significantly impaired since higher rates of dead oocytes and DNA fragmentation and lower rates of viable, matured and fertilized oocytes were observed. The percentage of cleavage, blastocyst rates and cumulus cell parameters (viability, active mitochondria and GSH/ROS ratio) were also decreased. Moreover, the preservation of ovaries in medium TCM199 had a detrimental effect on cumulus cells and oocyte competence. In conclusion, ovary transport times up to 5 h in saline solution are the most adequate storage conditions to maintain oocyte quality as well as developmental capacity in sheep. A strategy to rescue the poor developmental potential of stored oocytes will be necessary for successful production of high-quality embryos when longer ovarian preservation times are necessary.

## 1. Introduction

Assisted reproduction technologies (ARTs) in small ruminants, such as sheep, have great potential for genetic improvement and dissemination programs, since they allow for a rapid and sustainable increase in animals of great genetic merit. Furthermore, ARTs are effective tools in the preservation of endangered species or breeds as well as in disease eradication programs [1].

Though the main lines of investigation in small ruminants have focused on germplasm banks and artificial insemination [2], over the past few decades, slight advances have been made toward the use of in vitro embryo production (IVP) [1,2,3]. Generally, improving the efficiency of IVP protocols in these species entails the use of ovaries of dead animals because a large number of samples should be collected. Unlike their in vivo counterparts, oocytes retrieved from dead animals exhibit reduced developmental potential [4]. Moreover, the slaughterhouses where adult animals are slaughtered are usually located in strategic places and often far from research laboratories. Storing ovaries for long periods of time due to long distances could compromise the viability of the oocyte. Considering that the quality of oocytes determines the developmental potential of embryos after fertilization [5], the preservation of oocyte integrity from the moment the animal dies until the ovaries are processed is of critical importance.

Immediately following death, the lack of blood flow prevents oxygen and energy supply and places the ovaries under ischemic conditions [6]. The main mechanism of injury in ischemia is hypoxia, and cells with high metabolic rates, including the ones that form the ovarian tissue, tend to be damaged very rapidly [7,8]. Acute hypoxia results in ATP depletion that triggers a switch to glycolysis, the major anaerobic pathway for ATP production [9]. ATP is broken down without being resynthesized, and eventually, decreased energy efficiency and accumulation of lactic acid produced by glycolysis also reduce intracellular pH, resulting in additional cellular dysfunction [6,9]. Notwithstanding the relationship between hypoxia/ischemia and organ damage is being established, the physiological mechanisms by which oocyte quality is affected with increased ovary storage time remain to be completely understood. It is therefore necessary to elucidate the events occurring in the oocyte at the cellular and molecular levels for the purpose of taking the appropriate measures to reduce this damage.

The goal of this study, therefore, was to evaluate the cellular and molecular events related to oocyte quality and developmental competence that occur throughout storage of sheep oocytes within ischemic ovaries after the death of the animal. This may help to create a better understanding of the mechanism of oocyte injury obtained from ischemic ovaries and to identify the temporal window for successful fertilization in IVP, particularly when ovary transport times are inevitably long. Ultimately, it will contribute to developing a strategy to reverse the poor developmental potential of stored oocytes within ovaries, which will have a great significance for ARTs.

## 2. Materials and Methods

The adult sheep ovaries were collected from an authorized slaughterhouse (“Ovinos Manchegos”), and sperm samples were obtained from the Germplasm Bank of the “Reproduction Biology Group”, which is officially authorized for collecting and storing semen from sheep (ES07RS02OC). All chemicals were acquired from Merck Life Sciences (Madrid, Spain) unless otherwise stated.

### 2.1. Oocyte Collection and In Vitro Maturation

Adult sheep ovaries (*n* = 1420) were obtained post-mortem and transported at 30 °C in physiological saline (8.9 gr/L NaCl) supplemented with penicillin (0.1 g/L) or at 38.5 °C in TCM199 medium supplemented with polyvinylpyrrolidone (PVA; 1 g/L), 4-(2-hydroxyethyl)-1-piperazineethanesulfonic acid (HEPES) (6.51 g/L), streptomycin (0.1 g/L), penicillin (0.1 g/L) and sodium bicarbonate (0.4 g/L) and were maintained for 13 h in the same media and at the same temperature. The mean age of animals was around 6 years old, and the sheep breeds were mainly Merino or mixed. Immature cumulus–oocyte complexes (COCs) were recovered by slicing the ovaries with a scalpel at 3, 5, 7, 9, 11 and 13 h post ovary collection. Then, a total of 4258 COCs from 8 replicates having a clear and homogeneous or moderate granular ooplasm and surrounded by at least three layers of tightly packed cumulus cells were selected and placed in TCM199 medium supplemented with HEPES (2.38 mg/mL), heparin (2 μL/mL) and gentamycin (4 μL/mL). In each replicate, the COCs of ovaries from the same treatments were mixed and homogeneously distributed. From those, 2327 COCs were mechanically denuded by vortex in phosphate-buffered saline (PBS) supplemented with 0.1% PVA (*w/v*; PBS-PVA) and oocytes were either directly analysed, fixed in 0.5% glutaraldehyde (*v/v*) and stored at 4 °C for terminal deoxynucleotidyl transferase mediated dUTP nick-end labelling (TUNEL) analysis or snap-frozen and stored at −80 °C for mRNA analysis. In addition, 1931 COCs, collected from the last 4 replicates, were matured, fertilized and cultured in vitro following the protocol by Sánchez-Ajofrín et al. [10]. Briefly, COCs were washed in TCM199-gentamycin (4 μL/mL) and randomly placed in four-well dishes containing 500 μL of TCM199 and 4 μL/mL gentamycin, 100 μM cysteamine, 10 ng/mL follicle stimulating hormone, 10 ng/mL luteinizing hormone and 10% fetal calf serum [11] under mineral oil (Nidacon, Gothenburg, Sweden) and an atmosphere of 5% CO_2_ at 38.5 °C with maximal humidity.

### 2.2. In Vitro Fertilization (IVF)

After approximately 22 h, COCs were partially denuded by gentle pipetting, divided into groups of 40–45 oocytes and placed in four-well plates containing 450 μL of synthetic oviductal fluid (SOF, Table S1), as described by Takahashi and First [12], with 10% oestrous sheep serum (ESS). Frozen-thawed spermatozoa were separated using a Percoll^®^ density gradient (45%/90%) from two rams and capacitated for 15 min at 38.5 °C in 5% CO_2_ with SOF and 10% ESS. Spermatozoa were subsequently co-incubated with the oocytes at a final concentration of 10^6^ spermatozoa/mL for 18 h at 38.5 °C in 5% CO_2_.

### 2.3. In Vitro Culture (IVC)

After 18 h post-insemination (hpi), presumptive zygotes were transferred to 25 μL IVC droplets (approximately one embryo per μL) containing SOF supplemented with 3 mg/mL of bovine serum albumin and cultured in a humidified atmosphere of 5% CO_2_, 5% O_2_ and 90% N_2_ in air until day 8 post-insemination (dpi). Cleavage rate and blastocyst yield were examined at 48 hpi and 6, 7 and 8 dpi, respectively. All expanded blastocysts were fixed in 0.5% glutaraldehyde (*v/v*) and stored for TUNEL analysis and cell-number evaluation.

### 2.4. Early Apoptosis Assay

**Figure 1:**
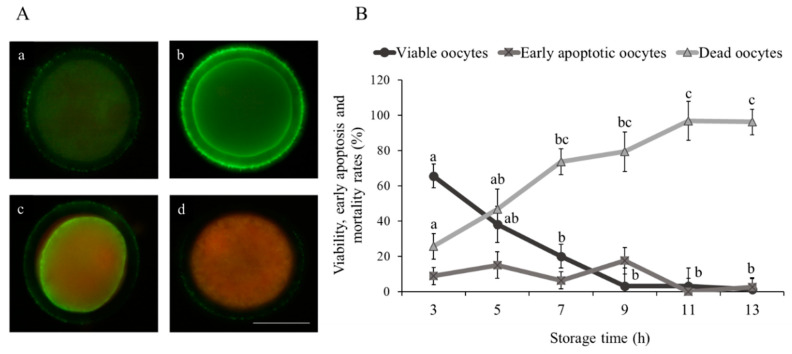
Effect of ovary storage time (3 to 13 h) on live/dead status and early apoptosis of immature sheep oocytes. (**A**) Representative images of sheep oocyte classification using Annexin-V staining: (a) viable oocyte, (b) early apoptotic oocyte and (c,d) dead oocytes. Scale bar = 50 µm. (**B**) Viability and early apoptosis rates (%): results are expressed as mean ± SEM. ^a,b,c^ Different letters indicate differences (*p* ≤ 0.05) among storage times.

To determine early apoptosis in oocytes, a total of 356 immature and denuded sheep oocytes were incubated in Annexin-V, fluorescein isothiocyanate (FITC) staining kit (Thermo Fisher Scientific, Barcelona, Spain) according to the manufacturer’s instructions. Briefly, oocytes were stained for 15 min with Annexin-V/FITC and 100 µg/mL propidium iodide (PI) at 37 °C in the dark. After incubation, oocytes were washed thrice in PBS-PVA and mounted on slides. Samples were evaluated at ×20 augmentation by fluorescence microscopy (Eclipse 80i, Nikon Instruments Europe, Amsterdam, The Netherlands) with the Intensilight C-HGFI module. The filter for excitation and the emitted fluorescence were EX 450-490 nm (DM 505; BA 520). Oocytes were classified into the following groups: early apoptotic oocytes (Annexin-V positive signal and PI negative signal; Figure 1A-a), viable oocytes (Annexin-V and PI signals were both negative; Figure 1A-b) and dead oocytes (Annexin-V and PI positive signals; Figure 1A-c and negative Annexin-V signal in the membrane and positive PI signal; Figure 1A-d).

### 2.5. Measurement of Glutathione (GSH) and Reactive Oxygen Species (ROS)

**Figure 2:**
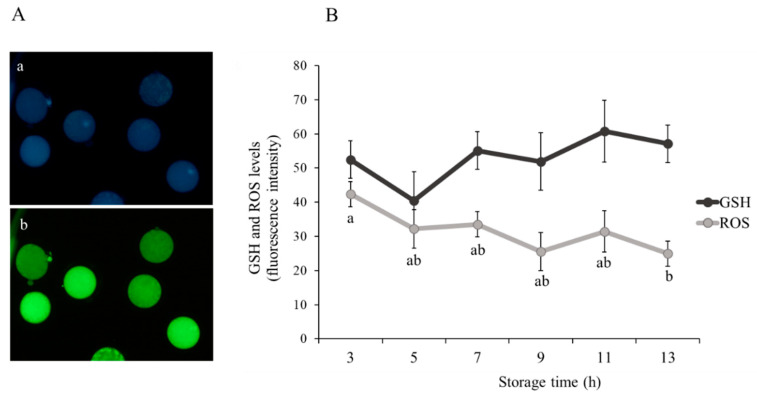
Effect of ovary storage time (3 to 13 h) on intracellular glutathione (GSH) and reactive oxygen species (ROS) levels of immature sheep oocytes: (**A**) representative images of intracellular (**a**) GSH and (**b**) ROS oocyte levels, scale bar = 100 µm, and (**B**) fluorescence intensity of GSH and ROS levels. Results are expressed as mean ± SEM. ^a,b^ Different letters indicate differences (*p* ≤ 0.05) among storage times.

A total of 350 immature oocytes were incubated in 50 µM Cell Tracker™ Blue (Thermo Fisher Scientific, Barcelona, Spain) and 10 µM of CM-H_2_DCFDA (Thermo Fisher Scientific, Barcelona, Spain) for 30 min at 37 °C in the dark to detect intracellular glutathione (GSH) and reactive oxygen species (ROS) levels, respectively. Oocytes were subsequently washed thrice in PBS-PVA and then placed on glass slides under cover slips. The fluorescence intensity was observed using ×20 augmentation by fluorescence microscopy (Eclipse 80i, Nikon Instruments Europe, Amsterdam, The Netherlands) and quantified using ImageJ 1.45s software (National Institutes of Health, Bethesda, MD, USA; Figure 2A-a GSH oocyte level and 2A-b ROS oocyte level).

### 2.6. DNA Fragmentation Assay

**Figure 3:**
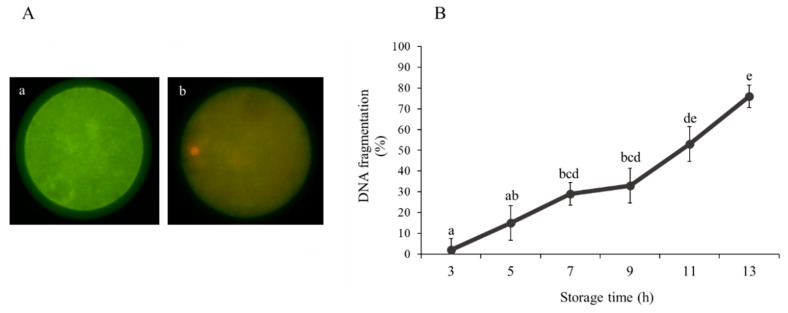
Effect of ovary storage time (3–13 h) on DNA fragmentation (positive terminal deoxynucleotidyl transferase mediated dUTP nick-end labelling (TUNEL) staining) of immature sheep oocytes: (**A**) representative images of (a) TUNEL-positive oocyte and (b) TUNEL-negative oocyte (scale bar = 50 µm) and (**B**) oocyte DNA fragmentation rates. Results are expressed as mean ± SEM. ^a,b,c,d,e^ Different letters indicate differences (*p* ≤ 0.05) among storage times.

The TUNEL method was used to detect DNA fragmentation combined with PI staining (oocytes) or Hoechst 33342 staining (blastocyst). Fixed immature sheep oocytes (*n* = 370) and blastocysts (*n* = 120) were permeabilized in 0.5% Triton X-100 in PBS for 1 h at room temperature. Next, In Situ Cell Death Detection Kit (Merck Life Sciences, Madrid, Spain) was used for the detection of DNA strand breaks in oocytes and blastomeres. According to the manufacturer’s instructions, samples were placed in 30 µL drops of TUNEL reagent with fluorescein isothiocyanate conjugated deoxyuridine 5-triphosphate (dUTP) and the enzyme terminal deoxy-nucleotidyl transferase and were incubated for 1 h at 37 °C. The positive control was incubated with DNAse (0.2 U/µL) at 37 °C in the dark for 1 h, while the negative control was incubated in the absence of enzyme terminal deoxynucleotidyl transferase. Immediately after, immature oocytes and blastocysts were washed three times in PBS-PVA and transferred onto slides in a drop of Slowfade™ with 6.25 µg/mL PI and 5 μg/mL Hoechst 33342 fluorescent dye, respectively. Samples were evaluated using a ×20 augmentation by fluorescence microscopy (Eclipse 80i, Nikon Instruments Europe, Amsterdam, The Netherlands). The DNA damage in oocytes was classified as TUNEL-positive (Figure 3A-a) and -negative (Figure 3A-b) according to fragmented cell nuclei. The DNA fragmentation in blastocysts was determined by the number of cells with fragmented nuclei (TUNEL-positive) in relation to the total cell number.

### 2.7. Measurement of Caspase-3 Activity

To monitor caspase-3 activity, 352 immature sheep oocytes were incubated for 30 min at 37 °C in 25 µL droplets of PBS-PVA containing 5 mM of PhiPhiLux-G1D2 (OncoImmunin Inc., Gaithersburg, MD, USA). After incubation, oocytes were washed twice in PBS-PVA and placed on slides under cover slips. Caspase activity was determined by fluorescence microscopy (Eclipse 80i, Nikon Instruments Europe, Amsterdam, The Netherlands), and intensity per unit area was quantified using ImageJ 1.45s software (National Institutes of Health, Bethesda, MD, USA; Figure S1).

### 2.8. Mitochondrial Membrane Potential Analysis

Membrane potential was determined by incubating 340 immature oocytes for 30 min at 37 °C in 0.5 µM of JC-1 dye (Thermo Fisher Scientific, Barcelona, Spain). After incubation, oocytes were washed twice for 5 min and then placed on glass slides. Oocytes were examined by ×20 augmentation by fluorescence microscopy (Eclipse 80i, Nikon Instruments Europe, Amsterdam, The Netherlands). Relative mitochondrial membrane potential was determined as the ratio of J-aggregate to J-monomer staining intensity with ImageJ 1.45s software (National Institutes of Health, Bethesda, MD, USA; Figure S2).

### 2.9. Assessment of Mitochondrial Distribution

Mitochondrial distribution patterns were examined by MitoTracker^®^ Red CMXRos (Thermo Fisher Scientific, Barcelona, Spain). At least 363 immature oocytes were incubated in PBS-PVA supplemented with 100 nM dye at 37 °C for 20 min. Oocytes were washed and then placed on glass slides and examined under ×20 augmentation by fluorescence microscopy (Eclipse 80i, Nikon Instruments Europe, Amsterdam, The Netherlands). Mitochondrial distribution was classified into two categories: abnormal mitochondrial distribution (Figure S3-d) in the cytoplasm and normal distribution (Figure S3a–c).

### 2.10. Quantification of Transcript Abundance

A total of 196 sheep oocytes were subjected to RNA extraction, complementary DNA (cDNA) synthesis and quantitative real-time PCR (qPCR) analysis as previously reported by Sánchez-Ajofrín et al. with minor modifications [13]. The RNA from groups of approximately 10 oocytes (3 replicates) was extracted using Dynabeads^®^ (Invitrogen, California, CA, USA) following the protocol by [14]. Briefly, oocytes were lysed at room temperature in 50 µL binding buffer for 5 min and hybridized with 10 µL magnetic beads for another 5 min. Then, samples were washed twice in 50 µL buffer A and twice in buffer B. Next, mRNA samples were eluted with 28 µL Tris-HCl. Following this, reverse transcription was carried out using the Fermentas™ First Strand cDNA Synthesis Kit (Thermo Scientific, Barcelona, Spain) in a total volume of 40 µL. After heating the samples at 65 °C for 5 min, cDNA was synthesized by adding 2 μL of reaction buffer (5x), 2 µL of dNTP Mix, 1 µL of RiboLock RNase Inhibitor, 1 μL of M-MuLV Reverse Transcriptase and 2.5 μL of nuclease-free water. Subsequently, reverse transcription reaction was performed by incubating for 5 min at 25 °C, followed by 60 min at 37 °C and 5 min at 70 °C.

After cDNA synthesis, PowerUp™SYBR^®^ Green Master Mix (Thermo Fisher Scientific, Barcelona, Spain) and a LightCycler 480 II system (Roche, Barcelona, Spain) were employed to determine the relative abundance of mRNA transcripts by qPCR. A final volume of 20 μL was reached by adding 10 μL master mix, 400 nM each of forward and reverse primers, 2 µL of cDNA template and nuclease-free water. The following PCR amplification conditions were used: 50 °C for 2 min, 95 °C for 2 min, 40 cycles of 95 °C for 15 s and 60 °C for 1 min. Immediately after, a melting curve analysis was performed to eliminate contamination by heating the samples to 95 °C for 5 s in a ramp rate of 4.4 °C/s, followed by 65 °C for 1 min with a heating rate of 2.2 °C/s and continuous fluorescence measurement. Each sample was analysed in duplicate, and reactions without any cDNA template (2 μL nuclease-free water) were used as the negative control.

The comparative cycle threshold and 2^−ΔΔCT^ methods [15,16] were used to calculate the relative transcript abundances of candidate genes: BCL2-associated X protein (*BAX*), BCL2 apoptosis regulator (*BCL2*), bone morphogenetic protein 15 (*BMP15*), caspase-3 (*CASP3*), fibroblast growth factor 16 (*FGF16*) and growth differentiation factor 9 (*GDF9*). Quantification was normalized against that of the endogenous control (Peptidylprolyl Isomerase A (*PPIA*)). Information on the qPCR primers is provided in Table S2.

### 2.11. In Vitro Maturation and Fertilization Assessment

After maturation, oocytes were stripped of the surrounding cumulus cells by gentle pipetting. To examine oocyte maturation and sperm penetration, cells were stained with Hoechst 33342 (1 μg/mL) for 10 min at room temperature, washed in PBS-PVA and then analysed with ×20 augmentation by fluorescence microscopy (Eclipse 80i, Nikon Instruments Europe, Amsterdam, The Netherlands). Maturation rate was defined as the number of oocytes with an evident polar body and metaphase II (MII) plate relative to the total number of oocytes analysed. Oocytes containing both female and male pronuclei (regardless of stage of decondensation) relative to the total number of oocytes matured were considered fertilized and were classified as normal (2PN) according to the number of swollen sperm heads and pronuclei in the cytoplasm.

### 2.12. Flow Cytometry Analysis of Cumulus Cells

Cumulus cells were collected from in vitro-matured oocytes and centrifuged at 12,000 rpm during 5 min. Pellet was resuspended in 125 µL PBS-PVA preequilibrated at 37 °C, and samples were stained and analysed by flow cytometry. To quantify cell live/dead status and apoptosis, samples were incubated with 10 µM YO-PRO-1 and 0.5 µM PI; for mitochondrial activity, cells were incubated with 200 mM of MitoTracker™ Deep Red (Thermo Fisher Scientific, Barcelona, Spain) for 20 min at 38.5 °C in the dark and then stained with 10 µM YO-PRO-1 and 0.5 µM PI; and for oxidative status (GSH and ROS levels), cells were incubated with 10 μM of Cell Tracker™ Blue (Thermo Fisher Scientific, Barcelona, Spain) and 10 μM of CM-H_2_DCFDA (Thermo Fisher Scientific, Barcelona, Spain) for 30 min at 38.5 °C to assess GSH and ROS levels, respectively, and subsequently stained with 0.5 µM PI. The percentage of YO-PRO-1−/PI− showed the proportion of viable cells, while YO-PRO-1+/PI− showed that of apoptotic cells. Viable cells with active mitochondria were represented by the percentage of MitoTracker+/YO-PRO-1−. Finally, oxidative status was measured by GSH and ROS production only in viable cells (PI−).

Cumulus cells analyses were conducted using a FlowSight^®^ imaging flow cytometer (Amnis, Merck-Millipore, Germany) equipped with violet, blue and red excitation lasers (405, 488 and 642 nm), 12 channels of detection and 10 available fluorescent channels. The system was controlled using INSPIRE^®^ software (v.3). The flow cytometer was calibrated daily using calibration beads according to the manufacturer’s instructions. A compensation overlap was performed before each experiment, and 1000 events were acquired per sample. In all cases, dot plots with aspect ratio and area were employed to exclude debris from cumulus cell populations and regions used to quantify cells subpopulation depended on the particular assay. The raw data were analysed using IDEAS^®^ software (AMNIS) and out of focus cells, debris and cell clumps were excluded from the analysis. 

### 2.13. Statistical Analysis

After determining that data were normally distributed and that variances were not heterogeneous, live/dead status, early apoptosis, DNA fragmentation, caspase-3 activity, GSH and ROS content, mitochondrial distribution and membrane potential, meiotic and fertilization competence, embryo production and total cell number were analysed by factorial ANOVA using the SPSS software (IBM, Armonk, NY, USA). For that, time of ovary storage (3, 5, 7, 9, 11 and 13 h) or type of ovary transport medium (TCM199 and saline solution) and the replicate (for IVM, IVF, embryo production, total number of cells and DNA fragmentation in blastocysts and cumulus cell analyses, four replicates were performed; for evaluation of oocyte viability and early apoptosis, DNA fragmentation, caspase-3 activity, GSH and ROS content, and mitochondrial membrane potential and distribution, eight replicates were performed) were considered fixed effects. Additionally, another factorial ANOVA was conducted to examine the relative abundances of mRNA transcripts, with time of ovary storage or type of medium and qPCR technical replicate (two replicates) as the fixed effects and the different target genes as the dependent variable. When a significant effect was observed, post hoc comparisons with Bonferroni correction were carried out. There was no evidence of statistically significant interactions between storage time and medium composition. Results are presented as mean ± S.E.M.

## 3. Results

### 3.1. Effect of Ovarian Transport Time on Oocyte Viability and Quality

As shown in Figure 1B, the percentage of viable immature sheep oocytes decreased (*p* < 0.05) from 7 h onwards (3 h = 65.62 ± 6.67% vs. 7 h = 20.00 ± 6.67%, 9 h = 3.12 ± 10.20%, 11 h = 3.12 ± 10.20% and 13 h = 1.25 ± 6.67%). Moreover, the lowest percentages (*p* < 0.05) of dead oocytes were observed at 3 and 5 h (25.62 ± 7.32% and 46.87 ± 11.18%, respectively) compared to 11 and 13 h (96.87 ± 11.18% and 96.25 ± 7.32%, respectively). Early apoptosis detected by phosphatidylserine localization using Annexin-V staining was not statistically different among groups (*p* > 0.05; Figure 1B).

A significantly higher level (*p* < 0.05) of ROS was recorded in immature sheep oocytes recovered from ovaries stored for 3 h (42.35 ± 3.69) compared to oocytes obtained from ovaries stored for 13 h (24.93 ± 3.69; Figure 2B). As also shown in Figure 2B, the different treatments did not exhibit different (*p* > 0.05) levels of GSH.

The number of immature sheep oocytes with TUNEL-positive fragmented DNA was lower (*p* < 0.05) at 3 h (1.94 ± 5.48%) of ovary storage, increased from 7 h (28.91 ± 5.48%) and recorded a maximum value at 13 h (75.88 ± 5.48%; Figure 3B).

The duration of ovary storage did not affect (*p* > 0.05) immature sheep oocyte caspase-3 intracellular activity (Figure S1), mitochondrial membrane potential (Figure S2) and mitochondrial distribution (Figure S3). Moreover, as shown in Figure S4, the relative abundance of mRNA transcripts of genes related to apoptosis (*BAX*, *BCL2* and *CASP3*) and oocyte quality (*BMP15*, *GDF9* and *FGF16*) did not show differences (*p* > 0.05) between 3, 7 and 13 h of ovary storage in immature sheep oocytes.

### 3.2. Effect of Ovarian Transport Time on the In Vitro Maturation and Fertilization Potential of Oocytes

The percentage of MII sheep oocytes recovered from ovaries stored for 3 h was higher (*p* < 0.05) compared to 7 and 13 h (Table 1. As expected, fertilization rate (2PN) was significantly increased after IVF in 3 h-derived oocytes compared to 7 and 13 h, with no significant differences between the latter groups (Table 1).

### 3.3. Effect of Ovarian Transport Time on Cumulus Cells from Cumulus–Oocyte Complexes (COCs)

**Figure 4:**
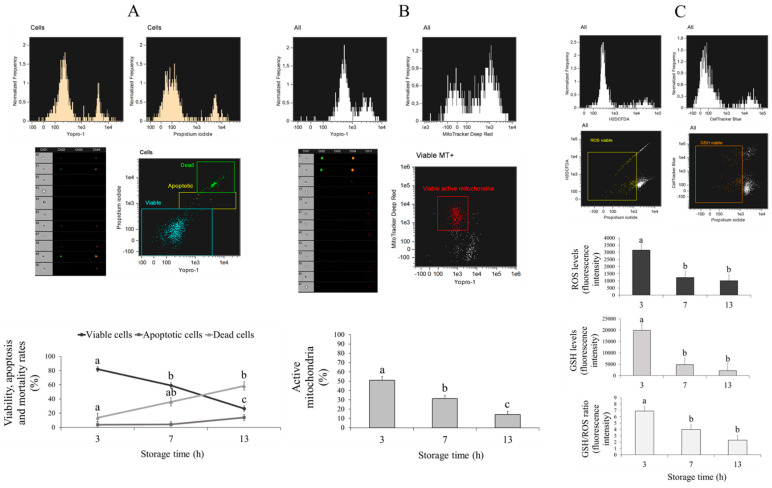
Effect of ovary storage time on (**A**) live/dead status and apoptosis, (**B**) percentage of active mitochondria and (**C**) intracellular GSH and ROS levels and the GSH/ROS ratio of cumulus cells collected from mature oocytes retrieved from ovaries stored for 3, 7 and 13 h: the results are expressed as mean ± SEM. ^a,b,c^ Different letters indicate differences (*p* ≤ 0.05) among treatments.

After maturation, COCs collected from ovaries stored for 3, 7 and 13 h were gently pipetted and detached cumulus cells were analysed by flow cytometry. Live/dead status, apoptosis, active mitochondria, and GSH and ROS levels were assessed. The results showed reduced (*p* < 0.05) cell viability as time increased (3 h = 82.06 ± 3.87% vs. 7 h = 59.16 ± 3.87% vs. 13 h = 26.53 ± 3.87%; Figure 4). Moreover, there was a lower (*p* < 0.05) percentage of dead cells at 3 h compared to 13 h (13.72 ± 6.08% and 58.50 ± 6.08%, respectively), although apoptosis did not show significant differences between storage time (*p* > 0.05; Figure 4).

Our results also showed reduced (*p* < 0.05) mitochondrial activity with increased storage time (3 h = 51.08 ± 3.78%, 7 h = 31.45 ± 3.45% and 13 h = 14.14 ± 3.45%; Figure 4). Furthermore, GSH and ROS levels and the ratio of GSH/ROS were higher after 3 h (GSH = 19890.59 ± 3044.95, ROS = 3153.65 ± 418.98 and GSH/ROS = 6.89 ± 0.78) of ovary storage compared to 7 h (GSH = 4813.30 ± 3044.95, ROS = 1234.67 ± 418.98 and GSH/ROS = 3.98 ± 0.78) and 13 h (GSH = 2213.70 ± 3044.95, ROS = 1009.18 ± 418.98 and GSH/ROS = 2.31 ± 0.78; Figure 4).

### 3.4. Effect of Ovarian Transport Time on In Vitro Embryo Development and Blastocyst Quality

The proportion of sheep oocytes that progressed to the first cleavage stage after IVF was significantly lower (*p* < 0.05) with increasing ovary storage time (Table 2. The percentage of total expanded blastocysts and percentage of blastocysts relative to the number of cleaved embryos was drastically decreased (*p* < 0.05) after 7 h of storage compared 3 h. Sheep blastocysts were not produced after 13 h of ovary storage (Table 2). However, the total cell number (3 h = 134.30 ± 6.01 and 7 h = 150.16 ± 12.02) and proportion of TUNEL-positive blastomeres (3 h = 13.02 ± 0.68% and 7 h = 12.34 ± 1.36%) in sheep blastocysts were similar (*p* > 0.05) between 3 and 7 h. 

### 3.5. Effect of Medium Type During Ovary Transport on Oocyte Developmental Competence and Quality 

The storage of sheep ovaries in TCM199 medium or saline solution did not show significant differences (*p* > 0.05) between the oocyte quality parameters studied, including oocyte live/dead status, apoptosis, caspase-3 intracellular activity, GSH and ROS levels, DNA fragmentation, mitochondrial distribution, mitochondrial membrane potential and relative mRNA transcript abundance (Figure S5).

The storage of ovaries with TCM199 resulted in lower (*p* < 0.05) rates of IVM, IVF, cleavage and blastocysts relative to the number of cleaved embryos (Table 3). Nevertheless, the total cell number of blastocysts was decreased (*p* < 0.05) as a result of storing the ovaries in saline solution (125.46 ± 6.72%) compared to TCM199 (159.0 ± 10.83%). Moreover, the number of total expanded blastocysts and TUNEL-positive blastomeres was similar (*p* > 0.05) in both media (Table 3).

Cumulus cells were also affected by the type of ovary storage medium. Thus, cumulus cells from ovaries stored in saline solution showed a greater percentage (*p* < 0.05) of viable cells and active mitochondria, while the TCM199 medium exhibited higher rates (*p* < 0.05) of dead cells (Table 4). The proportion of apoptotic cells and the GSH and ROS levels did not show significant differences (*p* > 0.05) between media (Table 4).

## 4. Discussion

In the present study, storage of sheep ovaries beyond 7 h had a detrimental effect on oocyte quality and subsequent development to the blastocyst stage. Similar results were obtained in rat ovaries where apparent histological changes were observed after 3 h of ischemia [17]. Notably, an in-depth analysis revealed that, after 7 h, oocyte live/dead status was dramatically reduced along with increasing storage time. After organ removal, an immediate consequence of the cessation of blood supply is the deprivation of oxygen and nutrients as well as the accumulation of metabolic waste, which may lead to cellular damage [18]. The crucial event is ATP depletion, which occurs within the first few minutes of oxygen stoppage [19]. This early event results in a transition from aerobic to anaerobic metabolism, which in turn leads to a rise in lactate and H^+^ levels that contribute in many mechanisms to cell injury related to ischemia [19]. In addition, the susceptibility of different types of cells to ischemic damage varies according to the degree of metabolic activity, and those with higher rates require a greater ongoing production of ATP [19]. For this reason, cells that form the ovarian tissue tend to be injured very rapidly by hypoxia [7,8].

Reduced viability of oocytes in post-mortem ovaries has been linked to degeneration of protein and DNA in horses [20] and domestic cats [21]. Remarkably, in the current study, we observed that the number of oocytes with fragmented DNA started to greatly increase after an ischemic time of 7 h, with no evidence of other oocyte apoptosis markers being significantly different (caspase-3 activity, phosphatidylserine binding by Annexin-V and mRNA transcripts). Moreover, there were no evident differences between storage times regarding mitochondrial membrane potential and distribution, which have been previously linked to many apoptotic stimuli [22,23,24]. Traditionally, apoptosis has been characterized by DNA damage as visualized by the TUNEL assay [25]. However, identification of terminal deoxy-nucleotidyl transferase (Tdt)–mediated deoxyuridine 5-triphosphate (dUTP) labelling in the nucleus of dying cells is not sufficient to demonstrate that cells are undergoing apoptosis, as the chromosomal DNA degradation and resulting DNA strand breaks also occur in necrotic cells [25,26]. Moreover, a pattern of TUNEL staining typical of necrotic cells was noticed in the present study with extensive staining of the cytosol, which may be due to the formation of large DNA fragments during karyorrhexis that are released into the cytosol upon nuclear disintegration [25,27]. 

Interestingly, intracellular ROS levels at 3 h were higher than at 13 h. It has been suggested that ROS quickly accumulate at the onset of ischemia despite limited O_2_ supply [28,29]. In the presence of xanthine oxidase (XOD) and O_2_, hypoxanthine can be converted to xanthine, which simultaneously produces superoxide anion (O_2_^•−^) [30]. During ischemia, the accumulation of XOD and hypoxanthine results in increased O_2_^•−^ production [31]. Considering that molecular O_2_ is the limited substrate in this reaction and that its availability decreases over time, it was not surprising to find that, after 13 h of ovary storage, intracellular ROS levels were significantly lower.

Successful embryo development will largely depend on the optimal accumulation of organelles, metabolites and maternal RNAs during oocyte growth [32]. In our study, the duration of ovary storage did not affect mitochondrial membrane potential, mitochondrial distribution and mRNA transcript of genes related to oocyte quality, although the embryonic yield was lower beyond 7 h of storage. A possible explanation could be that the assessments were performed on immature oocytes and that, in this cellular state, fluorescent dyes displayed less sensitivity to intracellular changes. Thus, different studies have shown contradictory data in relation to mitochondrial activity between immature and mature oocytes [33,34].

Besides, our results revealed that the number of oocytes showing MII and 2 PN after fertilization were lower when ovaries were stored longer than 7 h. Gaulden [35] suggested that hypoxic conditions could reduce oocyte intracellular pH, influencing the organization and stability of the meiotic metaphase spindle. Moreover, other researchers found that normal oocytes collected from under-oxygenated follicles showed chromosomal defects such as a compact arrangement on the MII spindle [36]. In our case, rather than chromosomal alterations, it is likely that the low rates of MII and 2PN are due to the high oocyte mortality following acute deprivation of oxygen after ovary collection. 

To gain further awareness of the biological consequences of prolonged transport time in stored oocytes, we examined cumulus cells and oocyte development in vitro. Cumulus cells play a critical role in oocyte maturation because they supply ions, metabolites and regulatory molecules that are necessary for meiotic progression, normal nuclear and cytoplasmic maturation of oocytes, and subsequent embryonic development after fertilization [37,38]. As expected, cumulus cells lost their supportive and protective functions when subjected to storage times longer than 7 h, since they showed decreased viability and impaired redox status and mitochondrial activity. Likewise, 7 and 13 h of ovarian storage resulted in drastic reduction in oocyte maturation, fertilization, cleavage and blastocyst rates. In fact, after 13 h of preservation, blastocysts were unable to develop. Therefore, reduced developmental potential of in vitro matured oocytes may also be related to impaired cumulus cell functions. Similar to our results, other studies have shown that the length of time that ovaries are held before oocyte recovery also affected developmental potential in several species. In sheep and pig, a delay of only 5–7 h reduced the maturation rate compared to that of oocytes placed immediately into maturation culture [39] or after 3 h of storage [6], respectively; in horses, 5–9 h had the same effects [40]. In addition, long-term storage (7–8 h) of ovaries reduced blastocyst formation rates after IVF in cattle [41] and intracytoplasmic sperm injection (ICSI) in horses [42].

Besides duration of ovarian storage, the type of medium where these organs are held plays an important role in determining appropriate transport conditions for oocyte survival and in vitro embryo development. Because TCM199 has more components (glucose, vitamins, amino acids and adenine sulphate) than saline solution and fully grown follicles are more metabolically active, we speculated that both follicles and oocytes may be better supported by the more complex medium. Although there were no differences between media for parameters used as indicators of oocyte quality, reduced oocyte developmental competence and cumulus cell quality were evidenced when ovaries were preserved in TCM199 medium. One possible explanation may be that ovaries from this group were kept at a higher temperature (38.5 °C) than the saline solution group (30 °C). In fact, preliminary results obtained by our group have indicated that the preservation of sheep ovaries in saline solution for 4 h at 38.5 °C negatively affects oocyte quality, IVM rates and cumulus cells compared to 30 °C (unpublished data). Moreover, it has been suggested that the use of low temperatures (4 °C) during long ovary storage times preserves oocyte quality possibly due to a decrease in cellular metabolism [43]. However, in our laboratory, oocytes from ovaries stored in saline solution at 4 °C for 3 h showed lower rates of MII after maturation (unpublished data), suggesting that low transport temperatures may have a detrimental effect during the transport of sheep ovaries. Differences with other authors such as Goodarzi et al. [44] could be related to the use of different transport media or even to the size of follicles from which the oocytes are obtained. More studies using a combination of different transport solutions, temperatures and storage times to retrieve the largest number of competent oocytes are necessary. 

## 5. Conclusions

Our study has provided new insight into the complex field of oocyte survival and in vitro development throughout ovary preservation. Moreover, it has contributed to understanding the effect of ovary storage in the physiological features of immature oocytes, which had not been evaluated up to date. We have demonstrated that transport ovary times up to 5 h in saline solution are the most adequate storage conditions to maintain oocyte quality as well as developmental capacity in sheep. After that, the quality and developmental potential of oocytes and cumulus cells dramatically decreases after storage of ovaries from 7 h. Based on these results, new strategies to evaluate the possibility of saving or rescuing the developmental potential of stored oocytes will be needed for successful production of high-quality embryos.

## Figures and Tables

**Table 1:** In vitro maturation and fertilization of sheep oocytes retrieved from ovaries stored for 3, 7 and 13 h.

Storage Time (h)	Total Oocyte (*n*)	Maturation MII (%)	Fertilization 2PN (%)
3	187	68.33 ± 6.20 ^a^	44.49 ± 4.47 ^a^
7	144	30.07 ± 6.53 ^b^	15.31 ± 4.70 ^b^
13	140	4.69 ± 6.53 ^c^	1.56 ± 4.70 ^b^

Data are expressed as mean ± SEM. The results represent four replicates. ^a,b,c^ Different letters indicate differences (*p* ≤ 0.05) among storage times.

**Table 2:** The effect of ovary storage time on rates of cleavage and blastocyst development in sheep.

Storage Time (h)	Total Oocyte (*n*)	Cleaved Embryo at 48 hpi (%)	Expanded Blastocyst (%)	
			Total	Cleaved
3	555	65.96 ± 5.23 ^a^	26.68 ± 2.19 ^a^	40.82 ± 4.58 ^a^
7	519	18.16 ± 5.23 ^b^	4.66 ± 2.19 ^b^	13.23 ± 4.58 ^b^
13	386	2.59 ± 5.23 ^b^	0.25 ± 2.19 ^b^	6.25± 4.58 ^b^

Data are expressed as mean ± SEM. The results represent four replicates. ^a,b,c^ Different letters indicate differences (*p* ≤ 0.05) among storage times.

**Table 3:** The effect of medium type during ovary transport on oocyte developmental competence and blastocyst quality in sheep.

Treatment	Total Oocyte (*n*)	Maturation MII (%)	Fertilization 2PN (%)	Cleaved Embryo at 48 hpi (%)	Total Expanded Blastocyst (%)	Expanded Blastocyst/ Cleaved (%)	TUNEL-Positive Blastomeres (%)
TCM199	934	28.27 ± 5.30 ^b^	13.64 ± 3.83 ^b^	23.81 ± 4.27 ^b^	9.14 ± 1.78	13.15 ± 3.74 ^b^	13.94 ± 1.22
Saline solution	997	43.46 ± 5.15 ^a^	27.27 ± 3.71 ^a^	34.00 ± 4.27 ^a^	11.93 ± 1.78	27.05 ± 3.74 ^a^	11.42 ± 0.76

Data are expressed as mean ± SEM. The results represent four replicates. ^a,b^ Different letters indicate differences (*p* ≤ 0.05) among media.

**Table 4:** The effect of medium type during ovary transport on cumulus cells in sheep.

Treatment	Viable Cells (%)	Apoptotic Cells (%)	Dead Cells (%)	Active Mitochondria (%)	GSH Levels (Fluorescence Intensity)	ROS Levels (Fluorescence Intensity)
TCM199	44.97 ± 3.87 ^b^	9.59 ± 3.29	45.07 ± 5.31 ^b^	26.42 ± 3.46 ^b^	8347.83 ± 2338.17	1591.14 ± 324.92
Saline solution	66.87 ± 3.87 ^a^	5.07 ± 3.29	27.08 ± 5.31 ^a^	38.97 ± 3.61 ^a^	9597.23 ± 2338.17	2007.19 ± 324.92

Data are expressed as mean ± SEM. The results represent four replicates. ^a,b^ Different letters indicate differences (*p* ≤ 0.05) among media.

## Supplementary Materials

The following are available online at https://www.mdpi.com/2076-2615/10/12/2414/s1, Table S1: Composition of solutions used in experiments, Table S2: List of primers used in qPCR of sheep immature oocytes, Table S3: List of abbreviations, Figure S1: Measurement of caspase-3 intracellular activity in sheep oocytes collected from ovaries stored for 3, 5, 7, 9, 11 and 13 h, Figure S2: Mitochondrial membrane potential in sheep oocytes collected from ovaries stored for 3, 5, 7, 9, 11 and 13 h, Figure S3: Mitochondrial distribution patterns in sheep oocytes collected from ovaries stored for 3, 5, 7, 9, 11 and 13 h, Figure S4: Relative mRNA transcript abundance pattern of genes of interest in sheep immature oocytes collected from ovaries stored for 3, 7 and 13 h, Figure S5: Live/dead status and apoptosis, GSH and ROS levels, DNA fragmentation, caspase-3 intracellular activity, mitochondrial membrane potential and distribution, and relative mRNA transcript abundance in sheep immature oocytes collected from ovaries stored with TCM or saline solution: the results are expressed as mean ± SEM.
