# Supplementary material for: Cellular and Molecular Events that Occur in the Oocyte during Prolonged Ovarian Storage in Sheep
Source: Animals (Basel). 2020 Dec 17;10(12):2414. doi: 10.3390/ani10122414 (PMC7766589; doi:10.3390/ani10122414)
Supplement: Supplementary file 1 [file animals-10-02414-s001.pdf]

animals

Supplementary materials: Cellular and Molecular Events that Occur in the Oocyte during Prolonged Ovarian Storage in Sheep

Alicia Martín-Maestro [1], Irene Sánchez-Ajofrín [1,*], Carolina Maside [1], Patricia Peris-Frau [1], Daniela-Alejandra Medina-Chávez [1], Beatriz Cardoso [1], José Carlos Navarro [1], María Rocío Fernández-Santos [1], José Julián Garde [1] and Ana Josefa Soler [1,*]

[1] SaBio IREC (CSIC-UCLM-JCCM), ETSIAM, Campus Universitario, s/n, 02071 Albacete, Spain; alicia.martinmaestro@uclm.es (A.M.-M.); carolina.maside@uclm.es (C.M.); patricia.peris@uclm.es (P.P.-F.); daniela.medina@uclm.es (D.-A.M.-C.); beacardoso_14@hotmail.com (B.C.); jnavarropedrosa@gmail.com (J.C.N.); mrocio.fernandez@uclm.es (M.R.F.-S.); julian.garde@uclm.es (J.J.G.)

* Correspondence: irene.ssanchez@uclm.es (I.S.-A.); anajosefa.soler@uclm.es (A.J.S.)

Received: 1 November 2020; Accepted: 13 December 2020; Published: date

Table S1. Composition of solutions used in experiments.

Solution	Compound	Concentration
Synthetic oviductal fluid (SOF)	NaCl	107.70 mM
	KCl	7.16 mM
	KH_2PO_4	1.19 mM
	$CaCl_2·2H_2O$	1.7 mM
	$MgCl_2·6H_2O$	0.5 mM
	$NaHCO_3$	25.07 mM
	Lactate	3.30 mM
	Pyruvate	0.30 mM
	Glutamine	200 mM
	Phenol red	spoon
	BME amino acids solution 50x	10 mL/500 mL
	MME non-essential amino acids solution 100x	5 mL/500 mL
	Penicillin	30 mg/500 mL

Table S2. List of primers used in qPCR of sheep immature oocytes.

Gene	Gene Function	Primer Sequence (5′–3′)	Product Size (bp)	Accession No.
PPIA	Reference gene	F- TCAACCCCACCGTGTTCTTC<br>R- GTCACCACCCTGGCACATAA	194	NM_001308578.1
BAX [a]	Apoptosis	F-GTTGTCGCCCTTTTCTACTTTGC<br>R-CAGCCCATGATGGTCCTGATC	89	NM_173894.1
BCL2		F-GGAGCTGGTGGTTGACTTTC<br>R-CTAGGTGGTCATTCAGGTAAG	518	NM_001077486.2
CASP3		F- GGAGTGAGATCCAGCAGAGC<br>R- TCCGTGTAAGATCGTTGTCTCT	125	XM_015104559.2
BMP15 [b]	Oocyte maturation	F-CTACGACTCCGCTTCGTGTGT<br>R-AGTGCCATGCCACCAGAAC	69	NM_001031752.1
GDF9 [c]		F-GAAGTGGGACAACTGGATTGTG<br>R-CCCTGGGACAGTCCCCTTTA	71	NM_174681.2
FGF16 [d]		F-CGCTTCGGAATTCTGGAGTT<br>R-TCCACTCCCCGGATGCT	62	NM_001192777.1

F, forward primer; R, reverse primer. [a–d] From Ferreira et al. [28].

Table S3. List of abbreviations.

Abbreviation	Definition
ARTs	Assisted reproductive technologies
BAX	BCL2-associated X protein
BCL2	BCL2 apoptosis regulator
BMP15	Bone morphogenetic protein 15
CASP3	Caspase-3
COCs	Cumulus–oocyte complexes
Dpi	Days post-insemination
ESS	Estrous sheep serum
FGF16	Fibroblast growth factor 16
GDF9	Growth differentiation factor 9
GSH	Reduced glutathione
Hpi	Hours post-insemination
IVF	In vitro fertilization
ICSI	Intracytoplasmic sperm injection
IVP	In vitro embryo production
IVM	In vitro maturation
MII	Metaphase II
$O_2^{\bullet -}$	Superoxide anion
PI	Propidium iodide
PBS	Phosphate-buffered saline
PPIA	Peptidylprolyl Isomerase A
PVA	Polyvinylpyrrolidone
HEPES	4-(2-hydroxyethyl)-1-piperazineethanesulfonic acid
qPCR	Quantitative real-time PCR
ROS	Reactive oxygen species
SOF	Synthetic oviductal fluid
TUNEL	Terminal deoxynucleotidyl transferase mediated dUTP nick-end labeling
XOD	Xanthine oxidase
2PN	2 Pronuclei

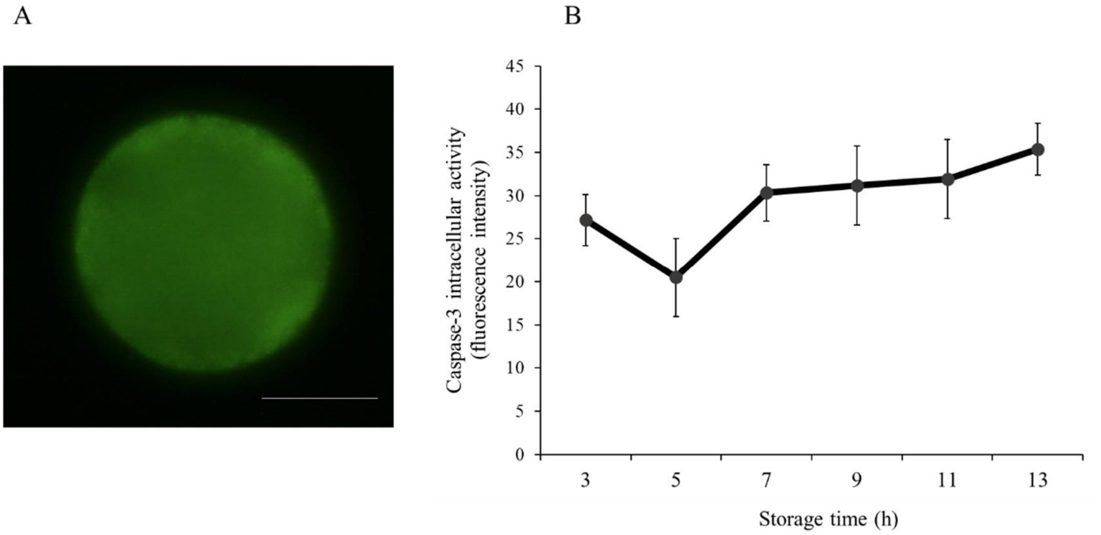

Figure S1. Measurement of caspase-3 intracellular activity in sheep oocytes collected from ovaries stored for 3, 5, 7, 9, 11 and 13 h. (A) representative image. Scale bar = 50 µm; (B) phiPhiLux-G1D2 dye fluorescence intensity per storage time. Results are expressed as mean ± SEM.

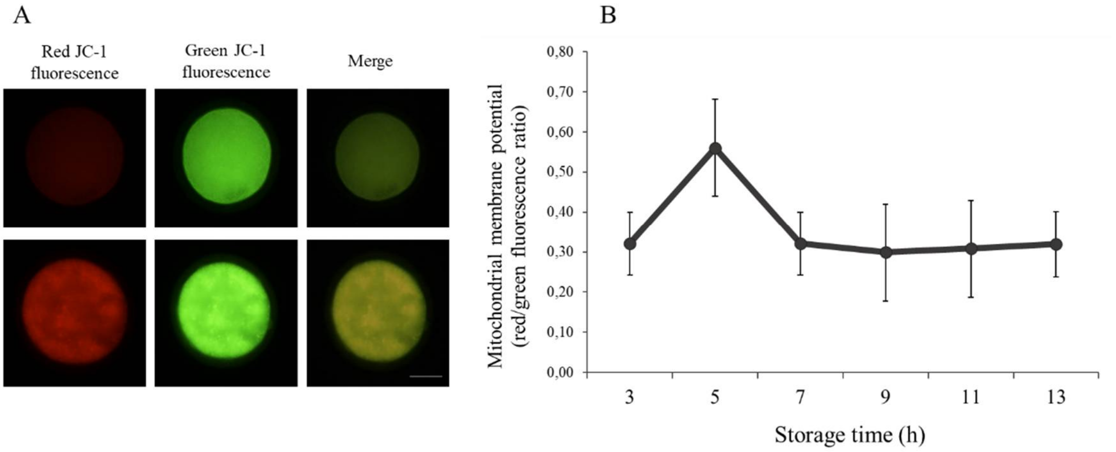

Figure S2. Mitochondrial membrane potential in sheep oocytes collected from ovaries stored for 3, 5, 7, 9, 11 and 13 h. (A) representative images. Scale bar = 50 µm; (B) ratio of red (J-aggregates; high membrane potential) to green (J-monomers; low membrane potential) fluorescence intensity per storage time. Results are expressed as mean ± SEM.

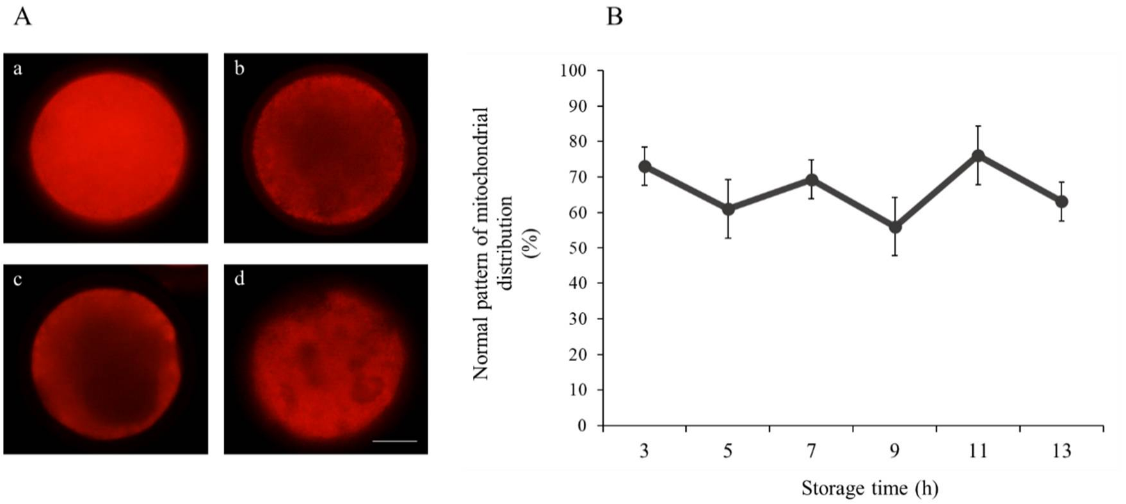

Figure S3. Mitochondrial distribution patterns in sheep oocytes collected from ovaries stored for 3, 5, 7, 9, 11 and 13 h. (A) Representative images: (a) Homogeneous distribution (b-c) Peripheral distribution (d) Abnormal distribution; (B) Percentage of normal pattern distribution (a-c) per storage time. Scale bar = 50 µm. Results are expressed as mean ± SEM.

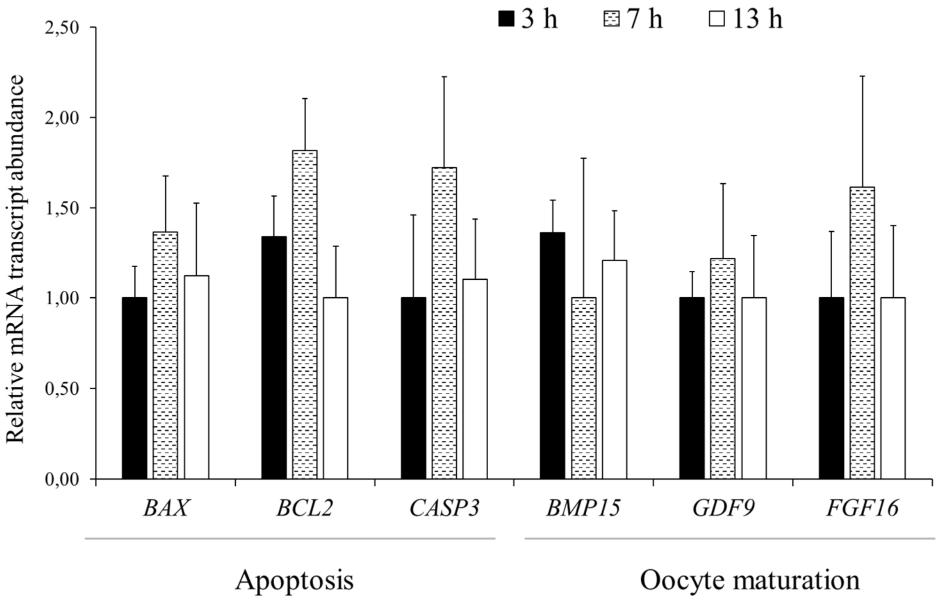

Figure S4. Relative mRNA transcript abundance pattern of genes of interest in sheep immature oocytes collected from ovaries stored for 3, 7 and 13 h. Results are expressed as mean ± SEM.

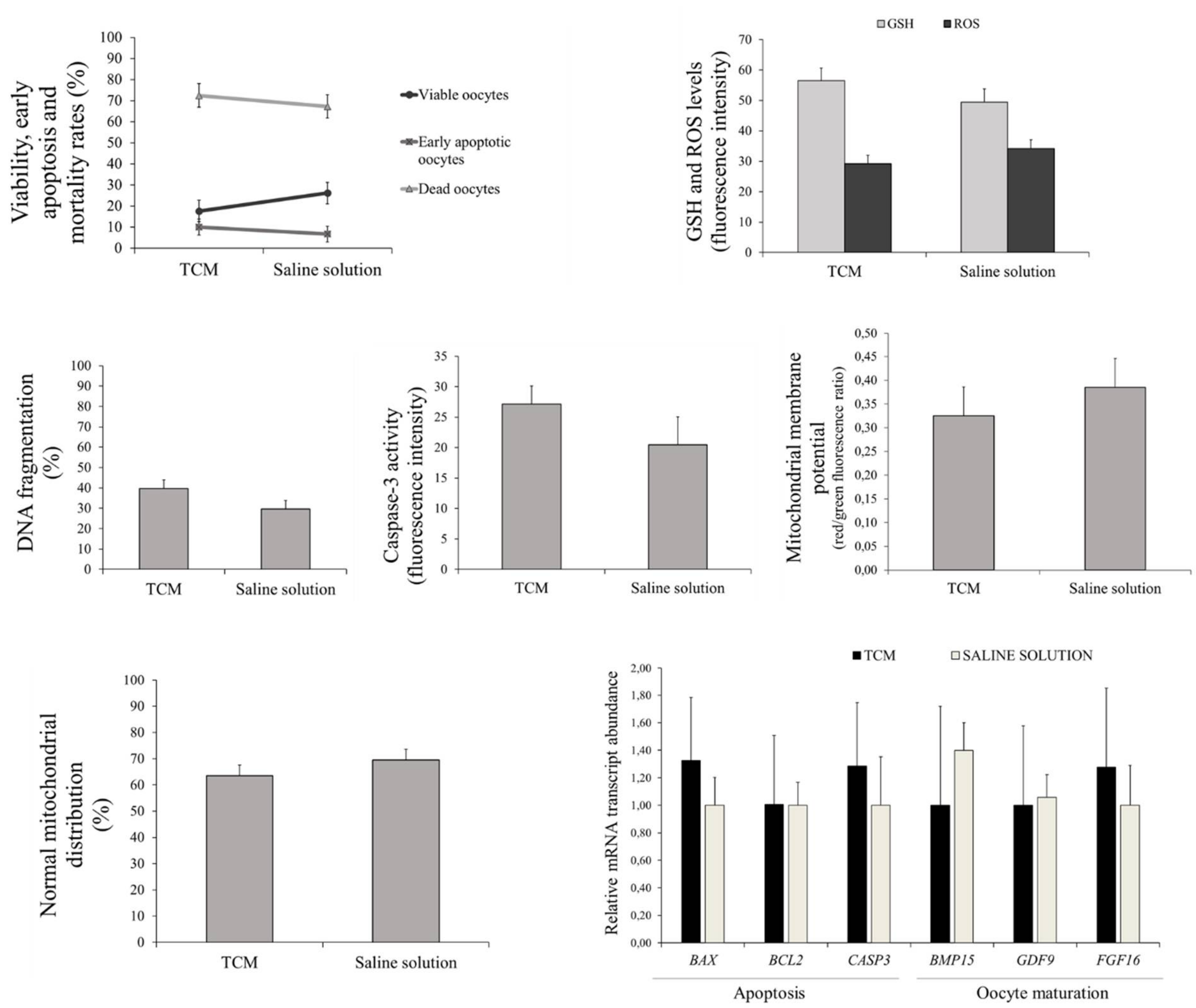

Figure S5. Live/dead status and apoptosis, GSH and ROS levels, DNA fragmentation, caspase-3 intracellular activity, mitochondrial membrane potential and distribution, and relative mRNA transcript abundance in sheep immature oocytes collected from ovaries stored with TCM or saline solution. Results are expressed as mean ± SEM.
